# Non‐orthogonal spectacle correction for irregular astigmatism

**DOI:** 10.1111/opo.13405

**Published:** 2024-10-12

**Authors:** Adela Hulpus, Ritchie Henry, Lynn White, Bernardo T. Lopes, Vito Romano, Ahmed Abass

**Affiliations:** ^1^ St Pauls Eye Unit Royal Liverpool University Hospital Liverpool UK; ^2^ Abergele Hospital Betsi Cadwaladr University Health Board Conwy UK; ^3^ Department of Research and Development LWVision Leicestershire UK; ^4^ Ophthalmology Eye Clinic Alder Hey Children's NHS Foundation Trust Liverpool UK; ^5^ Department of Medical and Surgical Specialties, Radiological Sciences, and Public Health, Ophthalmology Clinic University of Brescia Brescia Italy; ^6^ ASST Spedali Civili di Brescia Brescia Italy; ^7^ Department of Eye and Vision Science, Institute of Life Course and Medical Sciences University of Liverpool Liverpool UK; ^8^ Department of Materials, Design and Manufacturing Engineering, School of Engineering University of Liverpool Liverpool UK

**Keywords:** astigmatism, cornea, non‐orthogonal astigmatism, optical power

## Abstract

**Purpose:**

To investigate the potential improvement in visual acuity and subjective perception of image quality in patients with keratoconus using non‐orthogonal correction (NOC) cylinder trial lenses where the steep and flat power meridians are set at angles less or greater than 90°.

**Methods:**

A set of NOC plano/cylindrical trial lenses, where the axes between the power meridians were set at a range of non‐orthogonal angles, were used to refract 18 participants with keratoconus in whom 23 eyes were used for testing. Corneal elevation data were processed by bespoke MATLAB code from Pentacam Scheimpflug tomographer scans. Each participant first underwent subjective refraction using standard orthogonal cylinder trial lenses, and the monocular best‐corrected visual acuity (BCVA, logMAR) was recorded for each eye. They then underwent a second subjective refraction using NOC cylinder trial lenses created for the study and completed a questionnaire to elicit their subjective appraisal of letter clarity and ghosting.

**Results:**

Fourteen (61%) eyes demonstrated an increase in objective BCVA with the NOC versus the orthogonal correction; seven (30%) eyes showed no change and in two (9%) eyes, the BCVA was slightly worse. Further, 87% and 79% experienced an increase in letter clarity and a reduction in ghosting, respectively, independent of changes in BCVA. The majority of non‐orthogonal angles were in the range of 80°–85°, and it was possible to refine the cylinder and axis of the NOC further compared with the orthogonal correction. All but one of the participants said they would be interested in trying non‐orthogonal spectacles if the opportunity arose.

**Conclusions:**

Correcting irregular astigmatism in keratoconic individuals with non‐orthogonal spectacle correction may provide benefit in terms of increased visual acuity, improvements in letter clarity and a reduction of ghosting effects. This type of correction has the potential to improve the overall quality of life for patients with keratoconus.

**Key points**

- It is possible to improve visual acuity with non‐orthogonal spectacle lenses designed to correct irregular astigmatism in patients with keratoconus.
- Having experienced the potential improvement in visual acuity in the study, 96% of participants expressed interest in trying non‐orthogonal spectacles.
- This research marks a major milestone in improving the quality of life for patients with keratoconus.

## INTRODUCTION

Keratoconus is a progressive bilateral asymmetric condition of the cornea which results in biomechanical changes within the stroma, leading to thinning and corneal protrusion. As the condition progresses, the corneal shape and surface become more distorted, leading to a change from orthogonal to non‐orthogonal (irregular) astigmatism and an increase in refractive high‐order aberrations (HOAs).^1^ As conventional spectacle lenses employ orthogonal cylinder corrections, they cannot fully correct these types of refractive errors, leading to a reduction in visual acuity and symptoms of ghosting, doubling and reduced image contrast.^2^ Thus, patients have to turn to other methods of visual correction, for example, contact lenses, which aim to regularise the non‐orthogonal astigmatism or a range of surgical procedures, including corneal crosslinking (CXL), intracorneal ring segments (ICRS), topography‐guided surface ablations, and corneal grafting. Even after such surgical interventions, although vision and corrected visual acuity may improve, the patient may still be left with residual HOAs and irregular astigmatism.^3^ Even if these issues are reduced by contact lens wear, spectacles are still required as a backup when contact lenses cannot be worn. Additionally, contact lens wear presents a higher risk to the eye due to potential inflammation, infections and other complications such as scarring and lens intolerance. The ability to wear spectacles for everyday tasks, even in more advanced cases, would significantly improve the quality of life (QoL) for people with keratoconus and irregular corneas.^4^

### Astigmatism

Although past studies have examined the correction of HOAs in patients with keratoconus using adaptive optics and the changes to the neural adaptation that takes place within their visual systems,^5^, ^6^ the correction of the non‐orthogonal element of irregular astigmatism in spectacles has only had limited investigation.^7^ Regular astigmatism is a form of refractive error whereby light is refracted to two distinct focal points rather than one, as would be formed by a perfectly spherical surface. In regular astigmatism, the corneal and/or lenticular surfaces form an ellipsoid rather than a spherical shape. When these are simplified to quadric surfaces, the ellipsoid's principal meridians are always at right angles to each other, and accordingly the ocular flat and steep meridians are always assumed to be orthogonal. This assumption drives the current astigmatism measurement systems, which then imposes this orthogonality hypothesis on anterior eye measurement devices and systems. Perfectly regular orthogonal astigmatism, as described more than two centuries ago,^8^, ^9^ where the flat and steep meridians are positioned 90° to each other, is not the only type of astigmatism that exists, as even for normal eyes it is possible to find non‐orthogonal astigmatism.^10^ In the current standard arrangement, if the principal corneal meridians are non‐orthogonal in a particular eye, astigmatism is classified as irregular, and therefore is not correctable by standard sphero‐cylindrical lenses.^11^ This is routinely categorised as an HOA disorder, especially when a patient is diagnosed with keratoconus.

### Refraction of keratoconic eyes

When refracting the keratoconic eye, the usual objective clinical technique of retinoscopy is difficult, as irregular astigmatism causes ‘scissoring’ of the retinal reflex, although this in itself is a useful tool for diagnosis.^12^ Although an objective assessment of the keratoconic refractive state can be carried out using aberrometry, subsequent verification with subjective techniques can be difficult,^13^ as ultimately, the irregular astigmatism is being corrected with regular, orthogonal cylinders.

In terms of visual acuity, people with keratoconus have experienced normal visual acuity for a significant proportion of their lives, both with and without spectacles, and it is the comparison of their distorted vision with their remembered ‘normal’ vision that can make it difficult to adapt to an orthogonal spectacle correction^14^ with its attendant reduction in contrast and doubling and ghosting of images and letters. In addition, it is often difficult to quantify the vision of patients with keratoconus, as it is possible to read letters on a vision chart even when there is significant ghosting and doubling, thus giving a false impression of visual acuity.

The main aim of this study was to use subjective refraction techniques with orthogonal and non‐orthogonal cylinder trial lenses to ascertain if this type of correction could improve the visual experience of individuals with varying grades of keratoconus. An additional aim was to determine whether sphere, cylinder power and axis values would change between the orthogonal correction (OC) and non‐orthogonal correction (NOC).

## MATERIALS AND METHODS

### Subject numbers

In a pilot study,^7^ it was observed that the expected difference in visual acuity between non‐orthogonal and orthogonal lenses was a gain of 0.30 ± 0.20 logMAR. Considering a statistical power of 80% and α of 5%, the minimum sample size required to evaluate this difference was calculated as seven participants. Usually, a smaller change in visual acuity (2 lines or 0.20 logMAR) is considered clinically significant. To detect this minimal clinically significant difference the sample size required was 11. However, the study team decided to enrol 18 participants to ensure the reliability of the study and to account for any possible exclusion during follow‐up processes.

Eighteen patients were recruited from the Eye Clinic of the Royal Liverpool University Hospital in the UK, with the inclusion criteria of diagnosed keratoconus in at least one eye. Eyes were excluded if the topographical keratoconus classification (TKC) value, as determined by the Pentacam Scheimpflug tomographer (Oculus, pentacam.com), was designated as ‘normal’. Although keratoconus is a bilateral condition, it is often asymmetric and is often more advanced in one eye. As several of the individuals recruited for the study were newly diagnosed, some only had one eye that was considered keratoconic using the TKC value. No eyes were excluded as being too advanced for potential non‐orthogonal correction. Each participant was given an information sheet, giving details of the study so they could make an informed decision as to whether they wished to take part.

### Study participants

The London—Bromley's Research Ethics Committee approved the current study, which was conducted following the standards in the Declaration of Helsinki (21/PR/0561). Each participant underwent a tomography examination using the rotating Scheimpflug camera. They then underwent a subjective refraction using standard orthogonal cylinder trial set lenses followed immediately by refraction with the novel non‐orthogonal cylinder lenses. These plano/cylindrical trial lens sets were lathe cut from clear Perspex to fit 20 mm diameter trial set holders, similar to the lenses used in standard optometric trial sets. They were constructed to incorporate angles between the steep and flat meridians in steps of 5° from 60° to 85°. Cylinder powers ranged from −0.50 to −3.00 DC, and during testing, they could be combined to create a broader range of cylinder power corrections. Typical ophthalmic spherical trial set lenses were used in combination with the non‐orthogonal cylinders to perform the subjective refractions.

### Refractive routine—Orthogonal correction

So that the refractive routine was similar for both OC and NOC, routine tests for astigmatism that rely on orthogonality, such as Jackson cross cylinders or the astigmatic fan were omitted from both refraction routines. Where possible, the current refractive correction used by participants, or the most recent clinic refraction, was recorded and used as a starting point. Subjective refraction was then performed using a conventional ophthalmic trial set with spheres and orthogonal cylindrical test lenses, and the logMAR visual acuity was recorded. Each eye was refracted separately, and no binocular tests were carried out, although the participant was allowed to view the chart and the environment binocularly at the end of the refraction. The cylinder axis was assessed using a combination of subjective techniques whereby the cylinder lens was turned one way and another by the patient and then rechecked by the optometrist, and cylinder power was checked by increasing and reducing the cylinder power directly. Participants were asked to comment on the presence of any ghosting, doubling, or other visual distortions, and reminded that they would be asked about this in the post‐refraction questionnaire.

### Non‐orthogonal refraction

The non‐orthogonal refraction was then carried out immediately after the previous standard orthogonal subjective refraction; therefore, both refractions were carried out within a short time and by the same optometrist. As with the orthogonal refraction, each eye was refracted separately whilst the fellow eye was occluded. The first step was to replace the orthogonal cylindrical lenses with non‐orthogonal test lenses of the same power, starting with an angle between meridians of 85°, and visual acuity and participants' reaction to the effect on letter clarity and ghosting assessed. Cylinders with increasingly larger angles between meridians were inserted into the trial frame until it was clear that visual acuity was becoming worse.

The two non‐orthogonal corrections (NOCs) giving the best visual result were then manually optimised using simple addition and removal of powered spherical lenses, and the cylinder lens rotated to the position that gave optimal visual acuity and the least ghosting or doubling of letters. The combination providing the best logMAR VA and the least doubling and ghosting effects was recorded. This refractive process was repeated for the other eye, and then the participant was allowed to view the chart and surroundings binocularly, although no attempt was made to carry out a binocular vision assessment, as this was beyond the scope of this study to investigate the effect of NOC correction on binocular vision. The final non‐orthogonal prescription was written in the minus cylinder format: sphere/cylinder (non‐orthogonal angle) × axis. For example, −3.00/−1.50 (70) × 80 would mean that the cylindrical lens had meridians set at 70° instead of 90° and that the flattest meridian lay along 80°. Note that for non‐orthogonal lenses, flipping the lens over in front of the eye gives different results; a lens with a difference between meridians of 70° will present a difference of 110° when flipped the other way. Thus, the way in which the lenses are oriented in the frame is critical.

For the visual acuity assessment, an Early Treatment of Diabetic Retinopathy Study (EDTRS) chart was used. The EDTRS chart consisted of Sloan letters, had the same number of letters per row (5) with equal spacing of the letters and rows on a logarithmic (log) scale (the rows are separated by 0.10 log unit) and individual rows were balanced for letter difficulty. Visual acuity was measured in terms of both letters and lines. For this study, only distance acuity was considered.

After both refractive sessions had been completed, participants were asked to complete a survey to gather their subjective responses on letter clarity and ghosting, comparing the non‐orthogonal to orthogonal refractive correction. This was carried out utilising a Likert scale, where response options included ‘substantially better’, ‘moderately better’, etc., see Tables 1 and 2.

**TABLE 1:** Responses from the participants to questions concerning change in letter clarity with non‐orthogonal correction compared with changes in objective BCVA.

Letter clarity response	Grade	Frequency (%)	Change in BCVA
Substantially better	3	52	22%: No change in BCVA
			78%: Average change in BCVA was −0.19 ± 0.13 logMAR
Moderately better	2	22	20%: No change in BCVA
			80%: Average change in BCVA was −0.07 ± 0.08 logMAR
Slightly better	1	13	Average change in BCVA was −0.17 ± 0.10 logMAR
No change	0	4	Change in BCVA was 0.04 logMAR (1 eye)
Slightly worse	−1	0	NA
Moderately worse	−2	9	50%: No change in BCVA
			50%: Change in BCVA was −0.18 logMAR (1 eye)
Substantially worse	−3	0	NA

Abbreviations: BCVA, best‐corrected visual acuity; NA, not applicable.

**TABLE 2:** Responses from the participants to questions concerning change in perceived ghosting with non‐orthogonal correction compared with changes in objective BCVA.

Ghosting response	Grade	Frequency (%)	Change in BCVA
Substantially less	3	35	50%: No change in objective VA
			50%: Average change in VA was −0.24 ± 0.14 logMAR
Moderately less	2	26	Average change in BCVA was −0.14 ± 0.09 logMAR
Slightly less	1	22	40%: No change in objective BCVA
			60%: Average change in BCVA was −0.16 ± 0.12 logMAR
No change	0	17	25%: No change in objective BCVA
			75%: Average change in BCVA was −0.01 ± 0.04 logMAR
Slightly worse	−1	0	NA
Moderately worse	−2	0	NA
Substantially worse	−3	0	NA

Abbreviations: BCVA, best‐corrected visual acuity; NA, not applicable.

### Exclusions

Of the original 36 eyes, 13 were excluded from the results. The main exclusion criteria were applied to eyes regarded as normal using only the objective Pentacam TKC index classification to simplify the procedure. A broader criterion for defining keratoconus may be used in future studies, such as the Belin ABCD criteria.^15^ Sequential and already diagnosed patients with keratoconus were recruited from active clinic sessions, and the TKC value was used to eliminate participants who were unlikely to benefit from NOC. Eight eyes were excluded as being normal, two were excluded due to the irregular astigmatism being too high (>10.00 DC) to assess properly with the available non‐orthogonal trial lenses, two were excluded because the participant had a dry eye condition that made visual acuity measurement very unreliable and one was excluded due to potential amblyopia. Thus, the study included 23 eyes from 18 participants.

One participant (two eyes) had worn rigid gas‐permeable (RGP) contact lenses (for 40 years), and another wore a soft contact lens in only one eye. It was not practical to ask these participants to leave their contact lenses out before the study, as they would not be able to function normally, socially or at work. All other participants wore spectacles or relied on their better uncorrected contralateral eye. When comparing subjective refraction results, the RGP lens wearer was excluded from the analyses, as keratoconic eyes are vulnerable to the moulding effects of contact lenses. However, this participant's eyes were included in the analysis of visual acuity and the participants' own assessment of the impact of non‐orthogonal prescription on letter clarity and ghosting.

## RESULTS

The average age of the participants was 34.7 ± 12.9 years (range 19–67 years), and there were 12 males and six females. Of the 23 eyes included in the study, seven were from males and eight from females. Five eyes had an up‐to‐date spectacle prescription of 6 months or less and 10 had a current prescription from 6 to 12 months old. Four had a prescription ranging from 2 to 4 years old and four eyes had no previous prescription, either because the contralateral eye was essentially normal and the person preferred not to wear spectacles, or spectacles did not provide subjectively functional visual acuity. The eyes were assigned a keratoconus classification using the Pentacam TKC data, which allows a classification of five grades ranging from 0 for normal to 4 for severe keratoconus based on corneal biomechanical properties using machine learning (ML) algorithms.^16^, ^17^ Two eyes were classified as grade 1 with the ectasia as a result of previous laser‐assisted in situ keratomileusis (LASIK) surgery; two eyes as grades 1–2; five eyes as grade 2; three eyes as grades 2–3; 10 eyes as grade 3 and one eye as grades 3–4. Two eyes had undergone corneal collagen crosslinking (CXL), one of which had undergone LASIK (without CXL). There was a weak correlation (*r* = 0.12) between the age of the participants and TKC.

### Visual acuity and subjective assessment of vision quality

**FIGURE 1:**
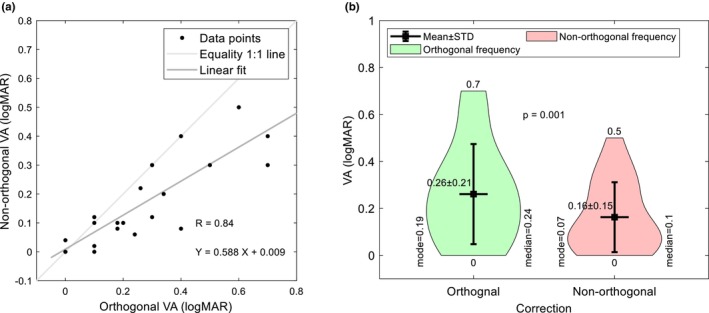
Orthogonal best‐corrected visual acuity (BCVA) in logMAR for each eye plotted against the BCVA (logMAR) achieved using non‐orthogonal correction. Panel (a) shows the correlation between the two parameters whilst (b) indicates their frequency distribution and significance. STD, standard deviation.

Figure 1a shows orthogonal best‐corrected visual acuity (BCVA) plotted against the BCVA with non‐orthogonal lenses for each eye and indicates that the NOC significantly improved BCVA (*r* = 0.84). A custom‐built violin plot, Figure 1b, showed that the most frequent orthogonal and non‐orthogonal BCVAs were 0.19 and 0.07 logMAR, respectively.

The BCVA found during the orthogonal refraction ranged from 0.70 to 0.00 logMAR for each eye, with the older participants tending to have poorer BCVA, despite there being a weak correlation between age and TKC. It was possible to determine a NOC for all 23 eyes, with 14 eyes (61%) achieving an improvement in BCVA. For the remainder, seven eyes (31%) demonstrated no change and two eyes (9%) showed a slight worsening of BCVA (logMAR). The average BCVA with OC was 0.24 ± 0.20 logMAR, which improved significantly to 0.16 ± 0.15 with the NOC (*p* = 0.001).

**FIGURE 2:**
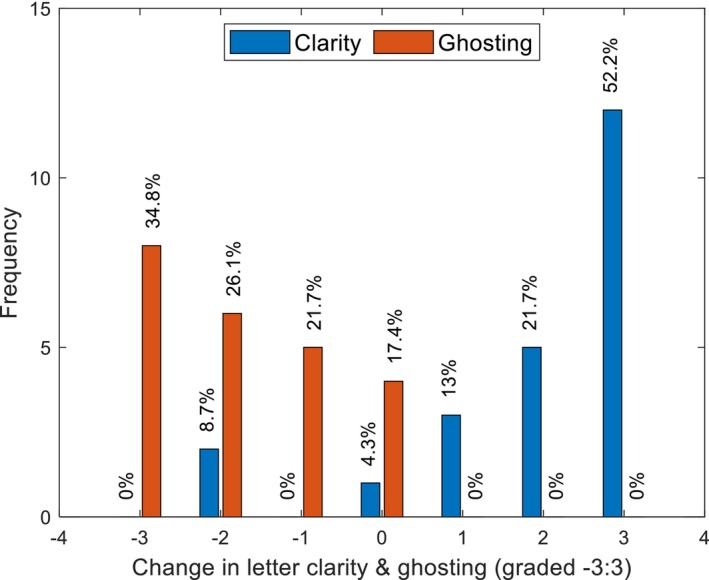
Distribution of the subjective experiences of subjects in terms of change in letter clarity and ghosting. The grading scale for letter clarity ranged from 3 ‘substantially better’ to −3 ‘substantially worse’, whilst ghosting followed the opposite pattern, with three indicating ‘substantially worse (increased ghosting)’ and −3 representing ‘substantially less’, that is, reduced ghosting. Both metrics had a neutral point at 0, signifying no change.

In addition to the objective measurement of visual acuity, participants were asked to comment in the follow‐up questionnaire on their subjective assessment of changes in the clarity of letters and any change in perceived ghosting. Figure 2 shows the frequency of subjective change in perceived letter clarity compared with the change in perceived ghosting. A total of 87% of participants experienced an increase in letter clarity with the NOC (Table 1), whilst 83% experienced a reduction in ghosting (Table 2). Interestingly, an improvement in perceived letter clarity or reduction in ghosting did not always correspond with objective improvement in BCVA. Conversely, objective BCVA improvements sometimes occurred without eliminating other visual issues, such as ghosting and doubling.

When participants were asked if, based on their experience, they would like to try non‐orthogonal spectacles if offered, 44% said they definitely would, 30% said they probably would, 9% were unsure, 13% did not respond and only one participant (4%) said they would not. Of the two participants who recorded a worsening of BVCA with NOC, one had keratoconus in both eyes, where one eye recorded slightly worse BCVA and the other slightly increased BCVA, and they felt overall a preference for the NOC. The other participant paradoxically did not record any subjective worsening of ghosting or reduction in letter clarity.

### Effect on refractive correction

**FIGURE 3:**
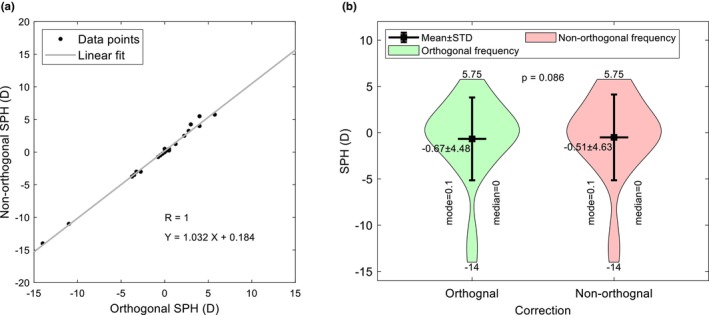
Comparison of sphere (SPH) powers between orthogonal and non‐orthogonal refractions. Panel (a) shows the correlation between the two parameters whilst (b) indicates their frequency distribution and significance. STD, standard deviation.

**FIGURE 4:**
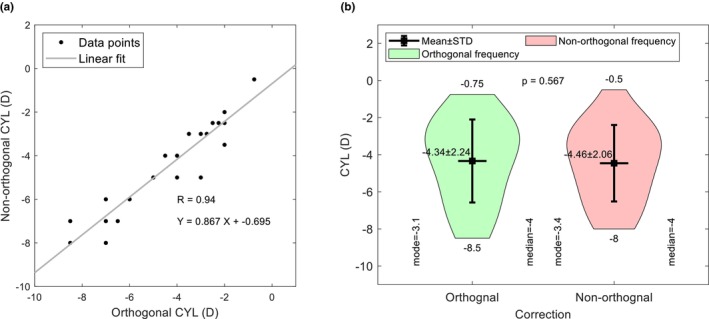
Comparison of cylinder (CYL) powers between orthogonal and non‐orthogonal refractions. Panel (a) shows the correlation between the two parameters whilst (b) indicates their frequency distribution and significance. STD, standard deviation.

**FIGURE 5:**
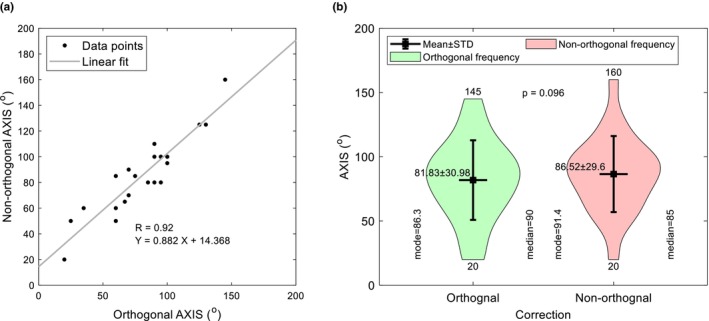
Comparison of cylinder axes (AXIS) between orthogonal and non‐orthogonal refractions. Panel (a) shows the correlation between the two parameters whilst (b) indicates their frequency distribution and significance. STD, standard deviation.

Subjective refraction relies on the patient responding to questions about whether a change in spherical power, cylinder power or cylinder axis changes the perceived clarity of optotypes on a test chart. As keratoconus distorts images over and above that caused by incorrect trial lenses, patients with this condition often resort to a ‘best guess’, which can result in unreliable manifest refraction results.^18^ The study investigated the possibility that if NOC enhanced letter clarity and/or reduced ghosting, thereby improving subjective responses, it might be possible to refine the sphere, cylinder and axis components of the final refraction. For this analysis, the RGP contact lens wearer (two eyes) was excluded from the analysis, as the moulding effect of the lenses was likely to affect the outcome. Figures 3, 4, 5 compare the sphere, cylinder and axis values, respectively, found with orthogonal and non‐orthogonal refractions. For 15 eyes (65%), there was no sphere change between the OC and NOC, whilst for the remainder, the sphere changes were very small, with only two eyes requiring a change exceeding ±0.50DS. Although nine (39%) eyes did not show a change in cylinder power, the remainder accepted changes ranging from −0.25 DC to −2.00 DC. In terms of the change in cylinder axis, six eyes (29%) showed no change in axis between OC and NOC, six eyes (29%) showed a change between ±5° whilst the remainder showed a change between 10° and 25°. Of those showing a change in axis, 65% were in a counter‐clockwise direction. In summary, refracting with non‐orthogonal cylinders appears to have very little impact on the spherical power, but refinement of cylinder and axis did occur.

### NOC angles

**FIGURE 6:**
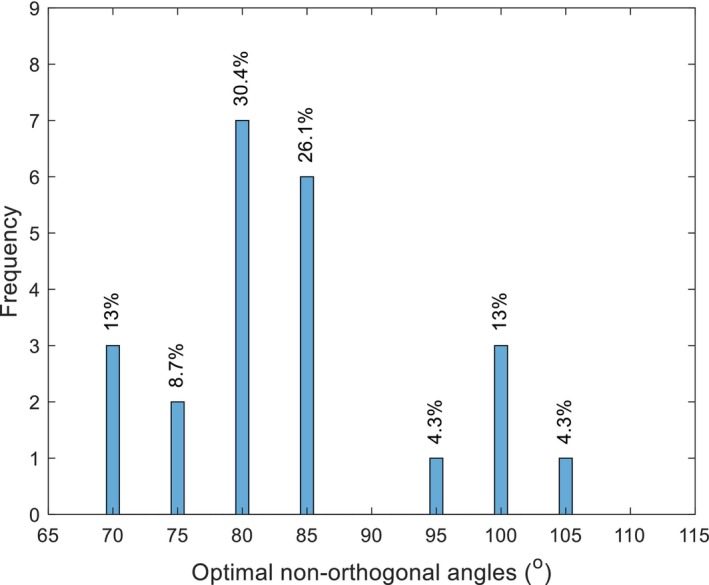
Frequency distribution of optimal non‐orthogonal angles between the cylinder meridians as determined by subjective refraction for all eyes.

The NOC angle represents the new angle between the steep and flat meridians in the astigmatic cylinder powers. Figure 6 shows the distribution of optimal NOC angles for all 23 eyes, showing that most (57%) were between 80° and 85°.

## DISCUSSION

Patients suffering from keratoconus experience a range of sensory inputs over time that differ from those with normal corneas. As the condition develops, the resulting corneal distortion increases exposure to various optical aberrations, and there is evidence that the visual system of such individuals undergoes neural adaptation, potentially reallocating sensory processing resources over a range of spatial frequencies.^19^

It has also been found that keratoconic eyes show less cardinally and rotationally symmetric sensitivities than normal eyes, which helps to compensate for the visual distortions induced by irregular astigmatism and HOAs.^20^ Thus, the expression of visual acuity in patients with keratoconus is a mix of optical and sensory output, and visual acuity measurement alone may not fully express the subjective visual experience of these individuals.

This small study is the first to look at correcting non‐orthogonal astigmatism in spectacle lenses and its effect on BCVA and subjective changes in their visual experience. Participants were recruited from patients attending routine clinical appointments and there was a wide range of severity and ages. Interestingly, although there was no correlation between age and severity as measured by TKC, there was a moderate correlation between age and presenting orthogonal BCVA. A limitation of the study is that slit lamp examination was not carried out to gather information about corneal haze and scarring, as this may well have been worse in older participants. With respect to carrying out non‐orthogonal refraction, there was only limited access to trial lenses with different non‐orthogonal angles and higher values of cylinder power. For this initial study, the aim was to investigate whether refracting with non‐orthogonal lenses would have any benefit or not, and future work could investigate expanding the range of lens powers and angles between meridians. The results show that objective improvements in BCVA were possible, together with subjective improvements in letter clarity and reduction in ghosting. However, it did not follow automatically that these would result in improved BCVA as recorded on a standard test chart. This would imply that the NOC reduced image distortion effects so that letters were more easily seen. Anecdotally, patients with keratoconus refracted with conventional lenses often struggle to define what is ‘better’ or ‘worse’ vision, often stating that letters are either ‘less ghosted but fainter’ or ‘clearer but more ghosted’.

There was a strong correlation between the presenting BCVA with the usual OC compared with the change in BCVA with the NOC. That is, the worse the original BCVA, the more improvement was seen with the NOC. The eyes with the least severe keratoconus experienced less change with NOC or found it slightly worse, suggesting those with more progressed conditions would find correction with non‐orthogonal cylinders beneficial.

### Refraction techniques

A previous study^7^ showed that participants with mild keratoconus responded well to NOC. The refractive technique was deliberately kept simple and based on low vision techniques for two reasons: first, to minimise differences in technique between subjects with varying severity of keratoconus, and second as best clinical practice in subjective refraction is based on assumptions that astigmatism is orthogonal, including many of the tests used to measure astigmatism. Therefore, an approach was adopted that would also minimise perceived differences between the two types of refractive techniques. As this was the first time non‐orthogonal lens correction had been used, there is no standardised methodology to draw on, as there is for orthogonal refraction. It was not clear at the outset how many attempts it would take to achieve the best possible outcome for the participant, and although it is possible that a better orthogonal refractive outcome may have been achieved with a more sophisticated technique, on balance, it was felt that it was more important not to fatigue the participants, so that contemporaneous results could be obtained from both types of refraction, especially as the participants were being asked to comment on differences in letter clarity and ghosting. However, it is a limitation of using this simple method of comparing orthogonal to NOC that the NOC was not compared with the best possible OC provided by more sophisticated techniques. Future work will examine how to improve non‐orthogonal subjective testing techniques and adapt orthogonal refractive routines to achieve a better comparison of the two types of refractive outcomes.

Whilst this method may not have been ideal for each individual participant; it was notable that some participants responded very quickly and easily to the non‐orthogonal refraction, whilst others found it more difficult. It is possible that in those cases, HOAs played a greater influence in causing letter distortion and ghosting effects. This simplified technique had limitations in that the non‐orthogonal angles were only available in 5° steps and smaller angles may have been more effective, especially in cases of higher astigmatism. Nonetheless, all 23 participants were able to determine a non‐orthogonal angle that gave either improved objective BCVA or a subjective improvement in letter quality.

It was notable that when moving from orthogonal to non‐orthogonal refraction, the cylinder power and axis could be refined further than with OC. Some participants were sensitive to changes as little as 0.25 DC, and for some, it appeared that once the optimal non‐orthogonal meridian angle was found, it was much easier for them to provide consistent subjective responses, due to reduced ghosting and increased letter clarity. Some of the cylinder changes were significant, with one being a −2.00 DC change.

### Non‐orthogonal angles

The majority of non‐orthogonal angles fell between 80° and 85°, and as the average cylinder power of the participants was −4.34 ± 2.24 DC, it is likely the combined effect of a relatively small deviation of orthogonality combined with the amount of astigmatism increased the visual disturbance. More work needs to be done with a greater number of eyes to investigate the range of non‐orthogonal angles found refractively in keratoconus and to potentially relate these values to the non‐orthogonality of the corneal shape. If it was possible to predict the optimal non‐orthogonal angle objectively, this would greatly facilitate non‐orthogonal refraction. The current preliminary study used the output from the Pentacam device, as it was based on earlier work examining the non‐orthogonality of the corneal surfaces. It was not possible at this point to find a link between non‐orthogonal angles on measured surfaces and the final, preferred subjective non‐orthogonal angle. Going forward, using aberrometry which considers all optical components would improve the chances of predicting non‐orthogonal angles objectively. Bell et al.^21^ successfully used wavefront aberration data to identify orthogonal sphero‐cylindrical refraction objectively that optimised the visual image quality for keratoconic eyes. This methodology could assist in identifying non‐orthogonal angles.

### Non‐orthogonal spectacles

A limitation of this study was that visual acuity and changes in visual disturbances were only considered for individual eyes, not for binocular vision, and limited to test lenses in a trial frame. It is not yet known how wearers of non‐orthogonal spectacles would adapt to using these in real life and how, in cases of monocular keratoconus, the wearer would adapt to a situation where one lens was orthogonal and the fellow lens non‐orthogonal. Even if on‐axis visual acuity with NOC showed a marked improvement over OC, this study did not have the opportunity to investigate off‐axis viewing, and there is the potential that this type of correction may result in a poor retinal image when utilised in spectacles. Further study of this possibility and investigation of whether monocular high astigmatism could be corrected fully due to the effects of aniseikonia is required to understand whether such lenses could be used practically in spectacle frames versus a trial frame refraction.

Nevertheless, correcting non‐orthogonal astigmatic errors in spectacles appears to have the potential for improving the QoL in patients with keratoconus and warrants further investigation.

## CONCLUSIONS

This study has shown that by utilising a range of non‐orthogonal cylinder lenses, significant improvements in the BCVA of patients with keratoconus can be achieved across a range of keratoconus severity, with the majority of angles between the power meridians found by subjective refraction lying between 80° and 85°. Subjective assessment showed that it was possible to improve the overall clarity of letters and reduce ghosting without necessarily improving the BCVA, and that this improvement could be achieved with a simple refractive routine using trial lenses. Further work incorporating NOC into spectacle lenses could assist in improving the visual experience of patients with keratoconus.

## AUTHOR CONTRIBUTIONS

**Adela Hulpus:** Data curation (lead); investigation (lead); methodology (supporting); project administration (supporting); validation (equal); visualization (equal); writing – original draft (equal). **Ritchie Henry:** Data curation (lead); investigation (lead); methodology (supporting); project administration (supporting); validation (equal); visualization (equal); writing – original draft (equal). **Lynn White:** Data curation (lead); formal analysis (lead); investigation (lead); methodology (equal); project administration (equal); validation (equal); writing – original draft (lead); writing – review and editing (lead). **Bernardo T. Lopes:** Formal analysis (equal); validation (equal); visualization (equal). **Vito Romano:** Data curation (equal); funding acquisition (equal); project administration (supporting); validation (equal); visualization (equal); writing – review and editing (supporting). **Ahmed Abass:** Conceptualization (lead); formal analysis (lead); funding acquisition (lead); investigation (lead); methodology (lead); project administration (lead); resources (lead); software (lead); supervision (lead); validation (equal); writing – original draft (lead); writing – review and editing (lead).

## FUNDING INFORMATION

This work was funded by Fight for Sight and the UK Keratoconus Self‐Help and Support Association, grant number 24KE20 (Principal investigator: Dr. Ahmed Abass).

## CONFLICT OF INTEREST STATEMENT

All authors of this article declare no conflict of interest.
